# Cure of Epithelioma of Sebaceous Origin by Resorcin

**Published:** 1890-12

**Authors:** W. Chasseaud


Cure of Epithelioma of Sebaceous Origin by Resorcin. By Dr. W. Chasseaud (*Bulletin Général de Thérapeutique*, Sept. 15, 1890. Page 207).

Under the title of Resorcin in Epithelioma of the Face, the *British Medical Journal*, of July 12, 1890, page 96, publishes the following: "Dr. Mario Luciani cites two cases of cutaneous epithelioma, in which he is said to have obtained a complete cure by the application of an ointment, composed of resorcin and vaseline. In one of these cases the patient, a woman of fifty-five years, had had a red nodule on her forehead for four years. The little tumor began to grow and ulcerate. The borders of the ulcer were indurated, and had a foul base. The disease progressing, and the patient not willing to submit to a surgical operation, the doctor applied to the ulcerated surface, after washing with a two per cent. solution of borax, an ointment composed of thirty grammes of resorcin and 100 grammes of vaseline. In the space of a month the ulcer took a healthy appearance, the borders softened, the sensation of heat and the lancinating pains disappeared. After three months of this treatment, the ulcer was completely cicatrized. The second case was a woman of sixty years, who for a year had suffered with a little tumor, situated upon the upper lip near the angle of the mouth, to the right. This tumor ulcerated in the same manner as in the preceding case. The same treatment was followed, with an equally happy result.

In the *International Journal of Medical Sciences*, VII., 1885, a case of epithelioma is reported by Dr. Rubin Antonio, in which a cure was obtained by the application of resorcin. The epithelioma, situated on the side of the nose, was about the size of a pea, and was accompanied by considerable hyperemia and infiltration of the skin. The neoplasm was washed twice daily with a solution of permanganate of potash, and the resorcin was applied in an ointment composed of fifteen parts, to twenty of vaseline, the tumor disappeared gradually, and at the end of five months there remained nothing but an insignificant cicatrix.

In May, 1886, I was called to see a case of epithelioma, situated on the right side of the nose, extending upward to the lower lid. Two years before, the woman had had a small tumor, which had ulcerated and had undergone several treatments for its removal, but without success. She had been seen by several physicians who pronounced it an epithelioma, and urged an immediate operation.

On showing the cases mentioned above, cured by resorcin, I insisted upon its being tried in this case. A microscopic examination presented all the characteristics of a lobulated epithelioma of sebaceous origin. At the end of a week the cancroid had increased somewhat in size, the neighboring parts had become ulcerated, but it had lost its granular aspect and presented an appearance of healthy tissue. The constant application of resorcin, which at first had been well borne, now began to produce severe pains. At the end of a month the patient refused to continue the treatment. The resorcin was suspended, and an ointment of oxide of zinc was applied. At the end of eight days, cicatrization began, and the whole ulcer was covered with healthy epithelium, save the left border toward the nose, and the upper border near the lower lid. The resorcin was again applied, and at the end of another month was succeeded by the oxide of zinc ointment. The same phenomenon was observed as before, *i. e.*, rapid cicatrization of the ulcerated parts. Since then I have repeatedly seen the patient, who has suffered no return of the disease.

Towards the end of the same year I saw a woman, about sixty years of age, who had been operated upon two years before, for an epithelioma of the left side of the nose, but without avail. She had applied, for a long time, ointments of boric acid, and of arsenate of soda; had been cauterized with nitric acid, with mercury, with the thermo-cautery, etc., the only result being an increase in the size of the tumor. I promised to cure her, and instituted the same treatment as in the preceding case. The same phenomena were reproduced, the ulceration increased at first under the influence of resorcin, and cicatrization took place rapidly under the oxide of zinc treatment. At the end of three months, she was completely cured, and has remained so until this day.

The third case of epithelioma which I have cured by resorcin, was situated on the right temple. Cicatrization was complete, but at the end of a short time a small point began to ulcerate; six months' treatment was necessary to obtain a radical cure, which still persists. Since then I have treated eight cases of the same kind with the same success, save in a single case. In all these cases I have observed that the extent of the ulceration produced by the resorcin, was in direct ratio to the extent of the infiltration. When the tumor is of recent origin, the resorcin causes it to disappear without enlarging it. In these last cases, I have abandoned the permanganate of potash wash, also the oxide of zinc ointment, and have replaced the latter with a simple ointment, with the same happy result. The unsuccessful case was a man about fifty years of age. The epithelioma was situated on the upper and anterior part of the nose, and had invaded the lower lid, completely destroying the caruncle and part of the lachrymal duct. Cicatrization had been obtained everywhere, except near the lachrymal duct, where the resorcin, on account of its irritation to the eye, had to be suspended. W. C. K.

---

A Few Pointers on the Method of Urethral Injection.—Never use a glass syringe. I give preference to Goodyear's rubber syringe, which has a bulb shoulder on its nozzle, thus preventing the nozzle from entering too far into the urethra. Warm the injection fluid before using. Do not overfill the urethra. Never stroke the under surface of urethra after injecting, as is advised in the books. Never use injections until the acute stage is passed. Never give injections which irritate or burn.—*MacConnell in Times and Register.*

